# Achalasia Cardia: A Case Series

**DOI:** 10.31729/jnma.8649

**Published:** 2024-07-31

**Authors:** Nibedita Chapagain, Nishob Adhikari, Bidur Prasad Acharya, Yugal Limbu, Roshan Ghimire

**Affiliations:** 1 Kathmandu Medical College and Teaching Hospital, Sinamangal, Kathmandu, Nepal; 2 Department of General, Surgery, Kathmandu Medical College and Teaching Hospital, Sinamangal, Kathmandu, Nepal

**Keywords:** *achalasia cardia*, *case reports*, *fundoplication*, *hyperthyroidism*, *manometry*

## Abstract

Achalasia cardia is a rare disorder that impacts the lower esophageal sphincter and esophageal body. Due to its wide range of symptoms, it can be difficult to diagnose. Here we report three cases of Achalasia Cardia during a period of 9 months. The first patient, an 18-year-old male, presented with dysphagia and was evaluated with barium swallow and high-resolution manometry revealing Achalasia Cardia. In the second case, a 37-year-old female had a prolonged diagnostic journey due to multiple comorbidities before a barium swallow finally revealed achalasia cardia. The third patient, a 47-year-old female was promptly diagnosed with barium swallow. All the cases were successfully treated with laparoscopic Heller's myotomy with anterior Dor's fundoplication. This case series highlights the potential for delayed diagnosis and the importance of early recognition, tailored diagnostic approaches, and the efficacy of surgical management.

## INTRODUCTION

Achalasia is a rare esophageal disorder characterized by impaired muscle function, resulting from the loss of inhibitory ganglion in the esophageal myenteric plexus, with an annual incidence rate of 0.5-1.2 cases per 100,000 individuals.^1-3^ Achalasia Cardia is diagnosed through esophageal manometry and often treated with Laparoscopic Heller Myotomy.^1,4^ It is well recognized that patients with achalasia frequently have concurrent thyroid diseases, but less frequently, as in our case, the co-existence of achalasia and hyperthyroidism necessitates careful study.^5^

In this case series, we showcase three instances of achalasia cardia, having diagnostic dilemmas, and were treated successfully with laparoscopic Heller's cardiomyotomy and Dor's fundoplication.

## CASE 1

**Figure 1:**
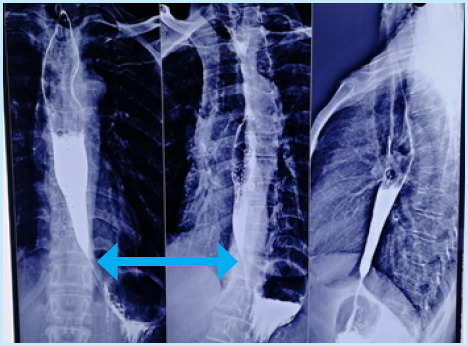
Barium swallow showing narrowing of the distal end of the esophagus with smooth dilatation of proximal end suggestive of a bird's beak appearance (double arrow).

An 18-year-old gentleman presented to the outpatient department of surgery with a complaint of difficulty in swallowing solid food for 10 months, which later progressed to difficulty in swallowing liquids as well associated with intermittent vomiting without any comorbidities. He underwent a barium swallow suggestingfeaturesofachalasia cardia andanesophago-gastro-duodenoscopy, revealing antral gastritis (Figure 1).

**Figure 2:**
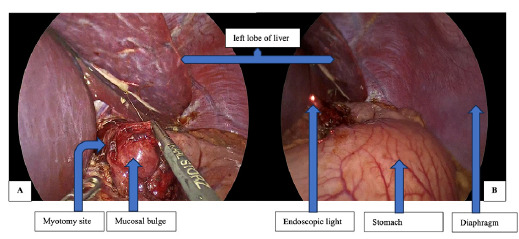
Intraoperative pictures; mucosal bulge (A) and visible endoscopic light (B).

Subsequently, high-resolution manometry (HRM) was done, which revealed type III achalasia cardia. The patient had previously undergone endoscopic balloon dilation but he did not experience any improvement in his symptoms indicating surgical management. The patient underwent laparoscopic Heller's myotomy with anterior Dor's fundoplication. Initial ly,with the retraction of the liver and elevation of the left lobe esophageal hiatus was visualized. The gastro-hepatic ligament was entered following the dissection of esophago-phrenic ligaments preserving the anterior vagus nerve. With the use of monopolar cautery, the muscle fibers are divided up to the cardia noting the bulge entering about 2-3 cm onto the stomach distally and 4-5 cm onto the esophagus proximally. Hemostasis was maintained and a check endoscopy was done intraoperatively which showed no leakage(Figure 2).

**Figure 3:**
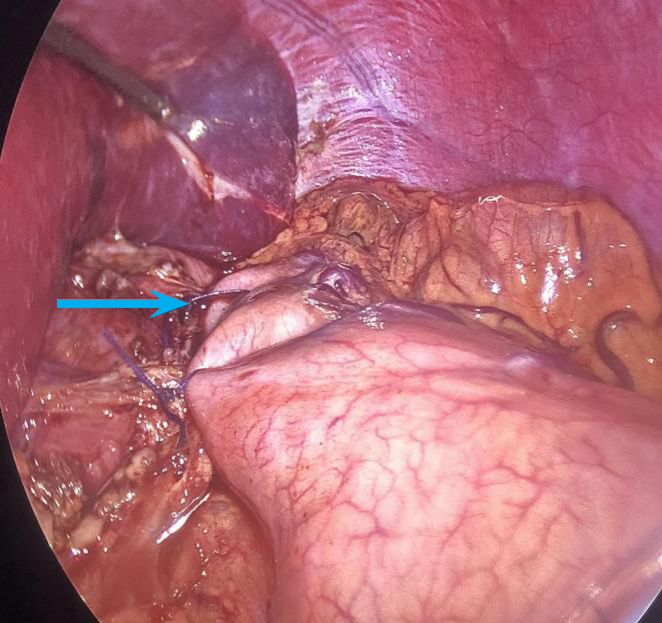
Anterior Dor's fundoplication (arrow).

Then, anterior Dor's fundoplication was performed using non-absorbable sutures (Figure 3).

The patient was discharged on the second postoperative day and followed up at regular intervals after the surgery which is done mostly on a clinical basis as per the symptoms and along with high-resolution manometry, barium esophagogram, and pH testing whenever required. Our patient showed significant improvement in his symptoms and continued to the next follow-up.

## CASE 2

**Figure 4:**
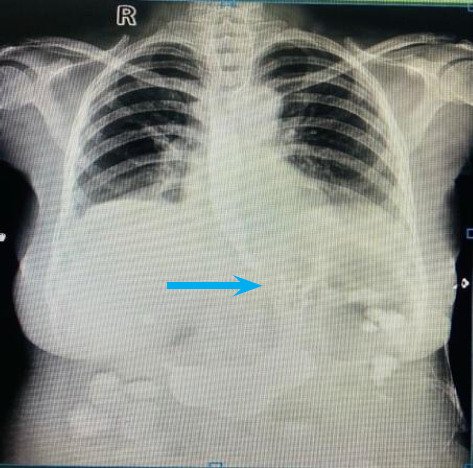
Barium Swallow showing narrowing of the terminal part of esophagus with smooth dilatation of proximal end (arrow).

A 37-year-old lady patient presented to the outpatient department of surgery with a history of difficulty in swallowing solids associated with intermittent recurrent vomiting, foreign body sensation at the throat, and non-documented weight loss for 1 year. The patient was a known case of schizophrenia and hyperthyroidism for the past seven years and one year respectively under regular medications. Her complaints led to several hospital visits which led to different incidental diagnoses like missed abortion and inclusion cyst. With that history on background suspecting possible esophageal motility disorder following an esophagogastroduodenoscopy, a barium swallow was performed which showed smooth tapering in the distal esophagus/gastroesophageal junction giving rat tail appearance with dilated esophagus thereby diagnosing Achalasia Cardia (Figure 4).

The patient was planned for Laparoscopic Heller's myotomy, and necessary preoperative investigations were performed. Her thyroid function tests showed abnormal results with anti-thyroid peroxidase at 178.7 IU/ml, FT3 at 4.45 pg/ml, FT4 at 23.9 pg/dl and thyroid stimulating hormone at 0.02 uIU/ml. Her erythrocyte sedimentation rate and C-reactive protein-quantitative were also high at 33 mm/hr and 3.5 mg/L, respectively. The patient was prepared for surgery and Laparoscopic Heller's myotomy with anterior Dor fundoplication was performed and 20 French abdominal drain was placed beneath the cardiomyotomy site and fixed to the skin. Her postoperative period was uneventful. Intraabdominal drain was removed on the 3rd postoperative day and she was started on a liquid diet which she was tolerating well. With a stable vital condition, she was discharged on the 3^rd^ postoperative day.

## CASE 3

**Figure 5:**
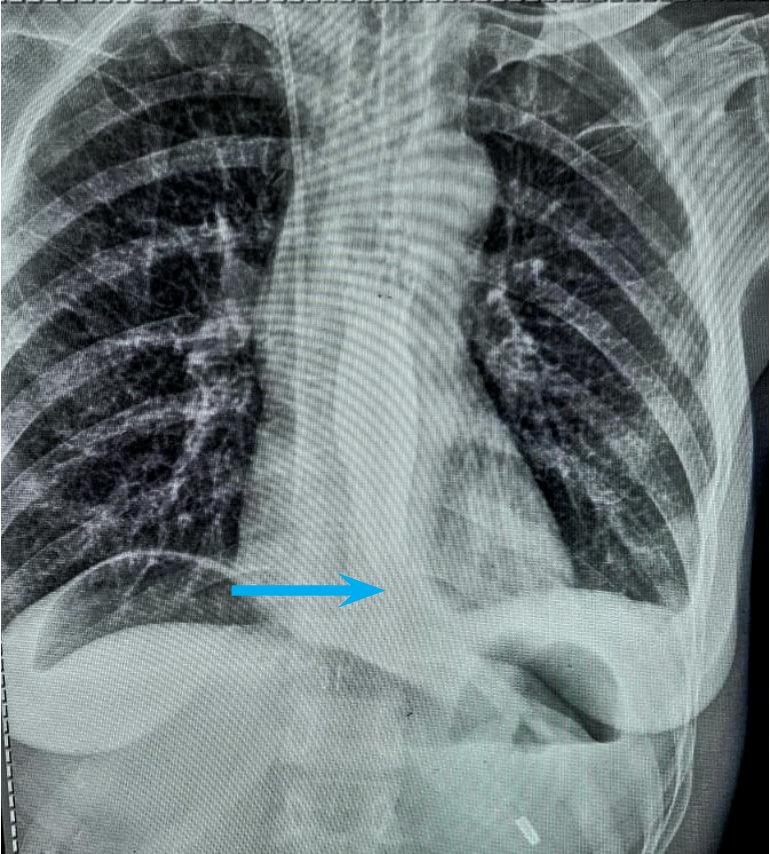
Narrowing of the distal esophagus with dilated proximal portion (arrow).

A 47-year-old lady presented with chief complaints of dysphagia for 3 months. Her physical examination was unremarkable. On performing a barium swallow, smooth narrowing of the lower end of the esophagus with gross dilatation of the proximal end suggestive of a bird beak appearance was noted (Figure 5). Due to unavailability of the high-resolution manometry the procedure could not be done in this particular patient.

With these findings, the patient was prepared for surgery and Heller myotomy with anterior Dor fundoplication was performed. Her intraoperative and postoperative periods were uneventful.

Liquid diet was initiated on the 2nd postoperative day and the patient was discharged on the same day. On follow-up, her symptoms had significantly improved and is being followed up as per protocol.

## DISCUSSION

Achalasia is a disorder that impairs the esophagus's ability to relax during swallowing and prevents peristalsis.^6^ Patients with achalasia cardia frequently report with symptoms like difficulty swallowing, chest discomfort, regurgitation of undigested food, and vomiting, which can result in unintentional weight loss.^7^ In the second case, the patient's history was difficult to ascertain due to her co-occurring schizophrenia, and her symptoms, which included vomiting and weight loss, were first attributed to her psychiatric condition and related drugs. But it took several hospital visits before she was ultimately identified as having Achalasia cardia during a barium swallow investigation. However, in the first case, the barium swallow and high-resolution manometry showed the typical features of Achalasia cardia, and the diagnosis was earlier. Similarly, our third case was diagnosed on the basis of barium swallow as well. Thus, for diagnosis, esophagogastroduodenoscopy, barium swallow, and high-resolution manometry arevital. Despite the findings of esophagogastroduodenoscopy and barium swallow, high-resolution esophageal manometry remains the gold standard for the diagnosis of achalasia cardia.^8^

The diagnosis of achalasia is suspected on the clinical grounds and barium swallow and esophagogastroduodenoscopy are performed as the initial diagnostic evaluation. For the confirmatory test, high-resolution manometry is key which provides detailed information on pressure measurement, subtypes and also rules out a few differentials. In some cases, we need to perform a computed tomography scan or magnetic resonance imaging to evaluate for any extrinsic compression or other abnormalities. The common differential diagnoses include esophageal stricture, esophageal malignancy, Chagas disease, diffuse esophageal spasm, scleroderma, nutcracker esophagus, pseudo-achalasia, etc.

Hypothyroidism is the thyroid ailment that is most frequently present in people with achalasia, which accounts for about 25% of cases. It is crucial to remember that even though it is uncommon, the coexistence of achalasia and hyperthyroidism needs to be carefully taken into account.^5^ In a prior study,^7^ out of 30 achalasia cardia patients had thyroid disease (four had hypothyroidism, two had hyperthyroidism, and one had just a thyroid nodule in the euthyroid condition).^9^ In the second case, the patient presented with hyperthyroidism as well and her anti-TPO level was also elevated. Monocyte infiltration into the myenteric plexus, the presence of the class II-Human Histocompatibility Complex DQwl antigen, and the presence of antibodies to myenteric neurons are all noteworthy discoveries that lend credence against the concept that an autoimmune mechanism underlies achalasia.^5^

The foremost goal of the currently available treatments, which can be classified as both medicinal and surgical, is to reduce the pressure placed on the lower esophageal sphincter to relieve the symptoms. Injections of botulinum toxin, endoscopic dilatation, and surgery are available options.^7^ Laparoscopically modified Heller cardiomyotomy is advised for achalasia individuals who do not have major comorbidities. Due to the reduction in symptoms, this surgery has a high percentage of patient satisfaction and a low risk of morbidity and mortality.^10^ Laparoscopic Heller's cardiomyotomy with Dor fundoplication was performed successfully in all cases.

The follow-up is equally crucial and should be done clinically and with the help of some important investigation modalities. Initially, after surgery, they should be monitored for immediate post-operative complications like perforation and infection and their ability to tolerate at least a liquid diet before discharge followed by wound healing. About a month after the surgery, their progress to tolerate soft diets from the liquid diet is an indication of improvement. Ideally, they should have a comprehensive evaluation with barium swallow at 3 months. The next evaluation is with the assessment of symptoms, nutritional status, barium enema, esophagogastroduodenoscopy, and high-resolution manometry in 6-12 months after the surgery. In long-term follow up their evaluation includes monitoring of complications like Barrett's esophagus and esophageal carcinoma with endoscopy as per protocol.

Thus, this uncommon disorder can be challenging to diagnose and has a wide range of symptoms. Fortunately, laparoscopic Heller's myotomy with Dor fundoplication has become a reliable and efficient treatment option, especially for patients with several comorbidities. For optimum outcomes, close monitoring and adherence to medications are necessary. Our case studies highlight the value of prompt action and adequate care in avoiding complications and raising patient quality of life.
